# Incidence, Remission and Mortality of Convulsive Epilepsy in Rural Northeast South Africa

**DOI:** 10.1371/journal.pone.0129097

**Published:** 2015-06-08

**Authors:** Ryan G. Wagner, Christian Bottomley, Anthony K. Ngugi, Fredrick Ibinda, F. Xavier Gómez-Olivé, Kathleen Kahn, Stephen Tollman, Charles R. Newton, on behalf of the SEEDS Writing Group

**Affiliations:** 1 Studies of Epidemiology of Epilepsy in Demographic Surveillance Systems (SEEDS)–INDEPTH Network, Accra, Ghana; 2 MRC/Wits Rural Public Health & Health Transitions Research Unit (Agincourt), School of Public Health, Faculty of Health Sciences, University of the Witwatersrand, Johannesburg, South Africa; 3 Epidemiology and Public Health Sciences, Department of Public Health and Clinical Medicine, Umeå University, Umeå, Sweden; 4 Department of Pharmacology and Clinical Neurosciences, Umeå University, Umeå, Sweden; 5 Department of Infectious Disease Epidemiology, Faculty of Epidemiology and Population Health, London School of Hygiene and Tropical Medicine, London, United Kingdom; 6 MRC Tropical Epidemiology Group, Faculty of Epidemiology and Population Health, London School of Hygiene and Tropical Medicine, London, United Kingdom; 7 Research Support Unit, Faculty of Health Sciences, Aga Khan University–East Africa, Nairobi, Kenya; 8 KEMRI/Wellcome Trust Research Programme, Centre for Geographic Medicine Research–Coast, Kilifi, Kenya; 9 INDEPTH Network, Accra, Ghana; 10 Neurosciences Unit, UCL Institute of Child Health, London, United Kingdom; 11 Clinical Research Unit, London School of Hygiene and Tropical Medicine, London, United Kingdom; 12 Department of Psychiatry, University of Oxford, Oxford, United Kingdom; St. Michael's Hospital, CANADA

## Abstract

**Background:**

Epilepsy is one of the most common neurological conditions globally, estimated to constitute 0.75% of the global burden of disease, with the majority of this burden found in low- and middle- income countries (LMICs). Few studies from LMICs, including much of sub-Saharan Africa, have described the incidence, remission or mortality rates due to epilepsy, which are needed to quantify the burden and inform policy. This study investigates the epidemiological parameters of convulsive epilepsy within a context of high HIV prevalence and an emerging burden of cardiovascular disease.

**Methods:**

A cross-sectional population survey of 82,818 individuals, in the Agincourt Health and Socio-demographic Surveillance Site (HDSS) in rural northeast South Africa was conducted in 2008, from which 296 people were identified with active convulsive epilepsy. A follow-up survey was conducted in 2012. Incidence and mortality rates were estimated, with duration and remission rates calculated using the DISMOD II software package.

**Results:**

The crude incidence for convulsive epilepsy was 17.4/100,000 per year (95%CI: 13.1-23.0). Remission was 4.6% and 3.9% per year for males and females, respectively. The standardized mortality ratio was 2.6 (95%CI: 1.7-3.5), with 33.3% of deaths directly related to epilepsy. Mortality was higher in men than women (adjusted rate ratio (aRR) 2.6 (95%CI: 1.2-5.4)), and was significantly associated with older ages (50+ years versus those 0-5 years old (RR 4.8 (95%CI: 0.6-36.4)).

**Conclusions:**

The crude incidence was lower whilst mortality rates were similar to other African studies; however, this study found higher mortality amongst older males. Efforts aimed at further understanding what causes epilepsy in older people and developing interventions to reduce prolonged seizures are likely to reduce the overall burden of ACE in rural South Africa.

## Introduction

Epilepsy, a common, chronic neurological disorder, affects 69 million individuals globally[1], contributing 0.75% to the global burden of disease[2]. Nearly 90% of this burden occurs in low- and middle- income countries (LMICs)[1]. On the African continent, where there are at least 10 million cases of epilepsy[3], population based-studies are needed to quantify the burden and inform policy.

Studies from LMICs suggest incidence[4] and mortality[5–7] rates are higher than in high income countries (HICs). Estimates of incidence from African studies range from 64 to 215/100,000 individuals per year[8–13], which is approximately twice the incidence found in HICs[4]. The higher rates in Africa might be due to increased incidence of risk factors, such as perinatal trauma and parasites[14], lack of adequate medical care or possibly differences between study methods[4,15,16].

The age-standardized rates of mortality in people with epilepsy (PWE) are 2 to 3 times that of the general population in HIC[17]. There are few estimates of mortality due to epilepsy in LMICs, including much of Africa, and generally these estimates are from small cohorts in areas with high incidence of epilepsy[18], which makes generalizing estimated mortality rates from these studies difficult. It is likely that PWE in LMICs experience higher mortality than the general population as well as PWE in HICs[6]. A recent study from rural Kenya estimated that the standardized mortality ratio (SMR), which measures mortality adjusted for the age structure of the population, in people with convulsive epilepsy was 6.5 (95%CI: 5.0–8.3)[7].

Few studies from Africa have explored risk factors for death in PWE. The study from rural Kenya found that non-adherence to anti-epileptic drugs, cognitive impairment and old age are risk factors for mortality[7]. A review of studies from Africa has suggested that epilepsy-related causes of death occur more frequently in PWE in LMICs than in HICs[19].

Population-based studies that estimate incidence and mortality and determine causes of death will provide researchers with a better understanding of epilepsy burden, which will allow targeted interventions to be developed. This large, population-based study seeks to estimate the incidence, remission and mortality of convulsive epilepsy in rural northeast South Africa. It also explores the causes of death in people with convulsive epilepsy.

## Methods

### Research Setting & Population

The study comprises two-cross sectional surveys conducted four years apart (2008 and 2012), in the rural Agincourt Health and Demographic Surveillance Site (HDSS) (http://www.agincourt.co.za) located 500 kilometers northeast of Johannesburg, South Africa. The Agincourt HDSS was established in 1992 as a platform for the collection of health and socio-demographic data in a rural setting to inform government policy[20].

Households are visited annually to capture vital events, including births, deaths and migrations. In 2008, the population stood at 83,121 in 25 research villages spread across 420 square-kilometers of semi-arid scrubland; subsistence farming and low-density cattle rearing are common. The majority of the population is Xi-Tsonga speaking and nearly one-third of the population is former Mozambican refugees who immigrated to South Africa as a result of the Mozambican civil war.

This highly mobile population is undergoing a rapid epidemiological transition marked by high levels of both communicable (HIV and TB) and non-communicable conditions (hypertension, diabetes and stroke)[21]. The HIV/AIDS pandemic has resulted in a substantial reduction in life expectancy; a 16-year reduction in females and 15 years in males between 1992 and 2006 (unpublished data). Life expectancy trends have improved since the mid-2000s with the rollout of anti-retroviral therapy at government clinics. The site contains six government clinics and two health centers, with three district hospitals 25–50 kilometers from the site.

### Identification of cohorts

In 2008, we screened 82,818 individuals (99.64% of the total 2008 population of 83,121) during a cross-sectional study to identify people with active convulsive epilepsy (ACE). We defined ACE as having ≥2 unprovoked convulsive seizures occurring more than 24 hours apart and ≥1 seizure occurring in the 12 months preceding the study or currently taking anti-epileptic drugs (AEDs)[14,22]. The methods used to ascertain cases are described elsewhere[22]. All individuals diagnosed with ACE in the cross-sectional study as well and those referred to the study team and diagnosed with ACE by the study clinician in 2008 were included in the ACE cohort.

We identified deaths occurring between 2008 and 2012 through follow-up within the annual census updates. In 2012, we repeated the cross-sectional survey. We identified individuals who had been present during the 2008 survey, and classified those from this cohort who were not present in the 2012 survey as out-migrated, untraceable or deceased. We contacted by phone individuals with ACE who had out-migrated. No effort was made to contact those in the general population (those without ACE at baseline) who had out-migrated due to financial and logistical constraints.

### Statistical Analysis

All data were entered into a mySQL database (OracleCorp, Redwood Shores, CA, U.S.A.) and were analyzed using Stata 13 (College Station, TX, U.S.A.).

### Incidence

All individuals experiencing an incident seizure were examined by the study neurologist (CRN) who performed a detailed clinical history that included previous medical conditions, including brain insults, and substance use to distinguish acute symptomatic seizures from unprovoked seizures. We defined an incident case of convulsive epilepsy as a person who had ≥2 seizures or a second unprovoked seizure between the date of the first cross-sectional study (1 August 2008) and the date of the second cross-sectional study (1 August 2012) without a known acute, underlying cause. Incidence was calculated by dividing the number of incident cases by the total number of person-years observed (pyo). Persons lost to follow-up, due to out-migration or death, were excluded from the denominator. We also present crude incidence in 5-/10-year age bands (S4 Table), and standardized incidence rates, which were calculated using the age distribution of the 2013 European standard population[23].

### Mortality

Age-specific mortality rates in both the epilepsy and general population were calculated for six age bands (0–5, 6–12, 13–18, 19–28, 29–49, and 50+ years). The mortality rate was estimated for both the general population and the epilepsy cohort by dividing the number of deaths by the pyo. Individuals with and without ACE in 2008 who out-migrated between 2008 and 2012 and were lost to follow-up contributed to the total pyo as they were known to have been living at time of out-migration. Additionally, people with ACE who had out-migrated between 2008 and 2012 and were confirmed, by phone, to be alive were included in the analysis. The standardized mortality ratio (SMR) was calculated by dividing the observed number of deaths in the epilepsy cohort by the expected mortality based on age-specific rates in the population without epilepsy. Additionally, an age-standardized mortality rate (standardized to the 2013 European standard population[23]) was also reported.

### Remission Rates & Duration

The DISMOD II (http://www.who.int/healthinfo/global_burden_disease/tools_software/en/) program was used to calculate age-specific rates of remission and duration of ACE based on the age-specific estimates of incidence, mortality and prevalence[22,24]. In the DISMOD model an individual is considered to be `free from epilepsy’ if they are not on AEDs and seizure free for one year. The proportion of those remitting per year was calculated using the formula: Remission Proportion = 1-exp(-rate per year). DISMOD models the expected duration of the disease based on the input prevalence, incidence and mortality parameters. The duration of disease is defined as the expected time an individual will live with epilepsy from the time of onset, taking into account both remission and mortality.

### Cause of Death

Cause of death (COD) was determined by verbal autopsy[25–27]. Trained lay fieldworkers systematically collected information on the characteristics and duration of symptoms and events up to the time of death from the individual closest to the deceased. Two clinicians independently reviewed the information and determined the COD, with a third independent clinician arbitrating non-consensus CODs. Deaths were ‘unclassifiable’ when the information provided by the respondent resulted in non-consensus after deliberation by the three clinicians. An independent neurologist (CRN) reviewed all cases for consensus. Additionally, CODs were categorized as being either directly (e.g. status epilepticus), indirectly (e.g. burns following a seizure) or unrelated to epilepsy. Deaths after prolonged seizures or without preceding illness (classified as sudden death epilepsy syndrome) were considered CODs directly related to epilepsy, while accidents during a seizure were considered indirectly related to epilepsy. Proportional mortality ratios (PMRs) were calculated by dividing the number of deaths in each COD category by the total number of deaths in the epilepsy cohort.

### Mortality Risk Factor Analysis

Poisson regression was used to explore the effect of 11 putative risk factors on mortality within the epilepsy cohort. These risk factors were either collected during the 2008 survey (age, sex, education, receiving income, human immunodeficiency virus (HIV) status, learning difficulties and age at onset) or collected during follow-up visits at 3-month intervals (current age, visit health facility, receiving treatment and seizure frequency), with the latter modeled as time-dependent covariates. Rate ratios adjusted for current age were estimated for all variables.

### Ethical Considerations

Written informed consent was sought from each participant in the study. Parental/guardian informed consent was sought in the case of children or patients with cognitive impairment. Ethical clearance for the study was received from the Human Research Ethics Committee of the University of the Witwatersrand (M120660) and the Mpumalanga Province Department of Health’s Research and Ethics committee.

## Results

Of the 82,818 individuals screened, 296 had ACE in 2008–245 were found using the three-stage method and 51 were referred to the study team from the community. The median age of those with ACE in 2008 and the general population was 27 years (interquartile range (IQR): 15–42.5) and 21 years (IQR: 10–35), respectively. The sex ratio (male/female) in the ACE cohort was 1.08 compared to 0.93 in the general population.

During the 4-year follow-up of 296 people with ACE, 33 individuals (11.1%) died and 27 (9.1%) out-migrated from the study area. The total person years observed (pyo) in the ACE cohort was 1122, with 49 pyo and 0 deaths contributed by those who had out-migrated. This resulted in a crude mortality rate of 29.4/1,000 pyo (95% Confidence Interval (95%CI): 20.9–41.4) (Table 1).

**Table 1:** Followed cohorts and outcomes of individuals and crude mortality rates, by age band, with and without ACE, Agincourt 2008–12.

**Agincourt HDSS Population 2008–12**						
**Age band in 2008**	**Number in cohort**	**Loss to follow-up**	**Person Years Observed**	**Deaths**	**Crude Mortality Rate (per 1,000)**	**95% CI**
***0–5***	11369	1779	41215	132	3.2	(2.7–3.8)
***6–12***	12908	1548	48063	59	1.2	(1.0–1.6)
***13–18***	10028	1208	37510	34	0.9	(0.6–1.3)
***19–28***	19629	2932	71461	354	5.0	(4.5–5.5)
***29–49***	18242	1675	67203	1016	15.1	(14.2–16.1)
***50+***	9580	365	35175	1222	34.7	(32.8–36.7)
***All Ages***	81756	9507	300627	2817	9.4	(9.0–9.7)
**Convulsive Epilepsy Cohort 2008–12**						
**Age band in 2008**	**Number in cohort**	**Out-migrations**	**Person Years Observed**	**Deaths**	**Crude Mortality Rate (per 1,000)**	**95% CI**
***0–5***	21	2	84	1	11.9	(1.7–84.6)
***6–12***	37	8	140	3	21.4	(6.9–66.2)
***13–18***	34	3	135	1	7.4	(1.0–52.6)
***19–28***	60	6	232	4	17.2	(6.5–45.9)
***29–49***	98	5	375	9	24.0	(12.5–46.1)
***50+***	46	3	155	15	96.7	(58.3–160.4)
***All Ages***	296	27	1121	33	29.4	(20.9–41.4)

During the 4-year follow-up of the 82,818 people screened, 2817 (3.4%) died, 9507 (11.5%) out-migrated and 1062 (1.3%) were untraceable. Those who out-migrated were found to be younger and female compared with those in the analysis cohort (S1 Table). The total pyo was 300 627 resulting in a crude mortality of 9.4/1,000 pyo (95%CI: 9.0–9.7).

### Incidence of Convulsive Epilepsy

Forty-eight incident cases of convulsive epilepsy were identified from those without convulsive epilepsy in 2008 that also took part in the 2012 survey. The median age of these cases was 24 years (IQR: 13–43) and the male/female ratio was 1.0. Those without ACE in 2008 who also took part in the 2012 survey contributed 276,400 pyo, and the incidence of epilepsy was 17.4/100,000/year (95%CI: 13.1–23.0). The highest rates were observed in the youngest (0–5 years) and oldest (50+ years) age bands (Table 2). The incidence in males was 17.7/100,000/year (95%CI: 11.8–26.4) and in females it was 17.1/100,000/year (95%CI: 11.4–25.5). Standardized to the age distribution of the 2013 European population, the incidence was 18.6/100,000/year (95%CI:13.3–23.8).

**Table 2:** Crude incidence of convulsive epilepsy by age band, Agincourt 2012.

Age band	Person-Years Observed	Incident Cases of ACE	Crude Incidence (per 100,000)	95% CI
***0–5***	12784	3	23.5	(7.6–72.8)
***6–12***	43844	9	20.5	(10.7–39.5)
***13–18***	32820	5	15.2	(6.3–36.6)
***19–28***	73644	10	13.6	(7.3–25.2)
***29–49***	73808	12	16.3	(9.2–28.6)
***50+***	39500	9	22.8	(11.9–43.8)
**Total**	276400	48	17.4	(13.1–23.0)

### Remission rate

The overall predicted remission rate was 4.6% (95%CI: 4.1–5.0) per year for males and 3.9% (95%CI: 3-4-4.5) per year for females (Table 3). Remission was highest in children under six years (16.0% and 30.5% per year in males and females, respectively).

**Table 3:** Duration and remission convulsive epilepsy using DISMOD II, Agincourt 2012.

Age band	Duration in years (95%CI)		Remission % per year (95%CI)	
	Males	Females	Males	Females
***0–5***	12.0 (11.3–12.7)	10.6 (10.11.3)	16.0 (14.8–17.1)	30.5 (28.5–32.6)
***6–12***	18.4 (17.3–19.4)	28.2 (26.7–29.6)	3.3 (3.1–3.6)	6.6 (5.8–7.3)
***13–18***	16.5 (15.5–17.5)	27.2 (25.8–28.7)	4.5 (4.2–4.8)	0.4 (0.2–0.6)
***19–28***	26.0 (24.6–27.4)	22.4 (21.3–23.6)	9.3 (8.8–9.8)	1.3 (0.9–1.7)
***29–49***	32.0 (30.1–33.8)	16.6 (15.7–17.5)	0.0 (0.0–0.1)	3.1 (2.8–3.4)
***50+***	25.3 (22.3–28.5)	14.2 (13.1–15.3)	2.0 (1.6–2.4)	6.5 (6.0–7.0)
***All ages***	21.5 (20.1–22.8)	19.7 (18.7–20.7)	4.6 (4.1–5.0)	3.9 (3.4–4.5)

### Duration of Epilepsy

Using the DISMOD II program, duration of epilepsy was predicted to be 21.5 years (95%CI: 20.1–22.8) for males and 19.7 years (95%CI: 18.7–20.7) for females (Table 3). Duration peaked in the 29–49 year age group in males, and in the 6–12 year age group in females.

### Mortality in people with ACE

Comparing the general population to people with ACE, the crude mortality rate ratio was 3.1 (95%CI: 2.1–4.2), whilst the SMR was 2.6 (95%CI: 1.7–3.5). Mortality rates were significantly higher in the 6–12, 19–28 and 50+ years age bands in the ACE cohort (Table 1) than in people without ACE, with the largest difference in the 50+ year age band (96.7 versus 34.7 per 1,000). The age-standardized (standardized to the 2013 European standard population) mortality in people with ACE was 67.1 per 1,000 (95%CI: 48.6–86.9).

### Cause of death from Verbal Autopsy

A verbal autopsy was completed for all 33 deaths within in the ACE cohort. Epilepsy was directly or in-directly related to 39.4% of deaths in the ACE cohort, communicable conditions contributed 36.4%, while chronic, non-communicable conditions (excluding epilepsy) accounted for 18.2% of the deaths. Approximately 6% of the deaths could not be classified (Table 4).

**Table 4:** Cause of death in people with convulsive epilepsy, Agincourt 2008–12.

Cause of Death	n	PMR %
*Directly related to epilepsy*		
Possible Status Epilepticus/ Prolonged Seizure	11	33.3%
Sudden unexpected death in epilepsy	0	
*Indirectly related to epilepsy*		
Injury/Accident/Self-harm	2	6.1%
*Unrelated to epilepsy*		
**Non-communicable**		
Underlying CNS condition	2	6.1%
Stroke	3	9.1%
Other	1	3.0%
**Communicable**		
Infectious Causes	12	36.4%
*Unclassifiable*	2	6.1%
**Total**	33	

### Risk factors for mortality in people with ACE

Older ages, being male, education and age at onset were all associated with mortality in a univariate analysis (S2 Table). Males had a higher risk of mortality after adjusting for age (Table 5).

**Table 5:** Age-adjusted rate ratios for mortality in people with convulsive epilepsy, Agincourt 2008–12

Factor	Deaths	Person-years observed	Rate Ratios (95% CI)		p-value
**Sex**					
Female	10	548	1	(n/a)	**0.013**
Male	23	574	2.6	(1.2–5.4)	
**Education**					
Yes	16	606	1	(n/a)	0.189
No	12	188	1.7	(0.8–3.8)	
**Receiving income**	** **				
Yes	3	80	1	(n/a)	0.773
No	25	678	0.8	(0.3–2.8)	
**HIV status**					
Negative	16	615	1	(n/a)	0.459
Positive	5	122	1.5	(0.5–4.0)	
**Learning Difficulties**					
No	22	794	1	(n/a)	0.18
Yes	11	292	1.6	(0.8–3.4)	
**Age at Onset**					
0–5	6	351	1	(n/a)	0.826
6–12	4	201	0.8	(0.2–3.0)	
13–18	2	111	0.8	(0.1–3.9)	
19–28	3	167	0.5	(0.1–2.3)	
29–49	10	188	1.2	(0.3–4.1)	
50+	6	58	1.3	(0.2–6.8)	
**Visits Health Facility^**					
Yes	23	776	1	(n/a)	0.472
No	7	248	1.4	(0.6–3.2)	
**Seizure Frequency^**					
Yearly	11	410	1	(n/a)	0.845
Daily	0	21	.	.	
Weekly or Monthly	21	598	1.2	(0.6–2.6)	
**Receiving treatment^**					
Yes	24	672	1	(n/a)	0.497
No	8	357	1.3	(0.6–2.9)	

^ time-varying variables

## Discussion

### Incidence

The incidence of convulsive epilepsy was estimated using two 3-stage cross-sectional surveys. In Kenya the sensitivity of the 3-stage survey was 48.6%[28]. Assuming similar sensitivity in South Africa, the incidence of convulsive epilepsy may be as high as 35.7/100,000/year (95%CI: 27.0–47.3).

In a review of the incidence of epilepsy, the median incidence was 81.7/100,000/year[4]. The incidence in our study was lower than this because we only considered convulsive epilepsy. In addition, risk factors for epilepsy are probably more common in other parts of Africa[22]. Further research to determine the incidence of all epilepsies is warranted.

Other African population-based studies have estimated similar incidences, when adjusting for the sensitivity of the methods used[8,11,12,29,30]. However, a recent study from rural Kenya found the incidence of convulsive epilepsy was 77.0/100,000 individuals/year, more than double the incidence in our study[13]. It is likely that the incidence is higher in Kenya because of higher levels of central nervous system infections and perinatal problems, which are important risk factors for epilepsy[14,31].

Incidence varied with age- the highest rates in the first 5 years of life and after the fifth decade of life. These findings are similar to the Kenyan study[13]. This age-incidence relationship differs from earlier African studies in which incidence declined over the lifespan[11,30]. The high incidence in the first decade of life (and most pronounced in the first 5 years of life in this study) may reflect genetic propensity, antenatal and perinatal infections or trauma. A family history of seizures and problems after delivery were previously found to be significant risk factors for ACE in Agincourt[22].

The high incidence in older ages, might reflect demographic and epidemiological transitions (marked by longer life expectancies and increased cardiovascular disease (CVD)[32], that are currently occurring in sub-Saharan Africa[33]. Within the Agincourt site, chronic CVD mortality is high, and is likely to increase further as people with HIV are treated and life expectancies continue to rise[34].

This study derives its findings from two cross-sectional surveys four years apart. The incidence of convulsive epilepsy may be underestimated as a result of the increased mortality rates observed 1–2 years after onset of seizures[17,35], and recall bias in cases where onset and remission occurred within the 4-year period between surveys. Additionally, individuals who experienced their first seizure long ago and their second seizure sometime between the two studies could have been classified as having had a single seizure. Finally, the open nature of the HDSS cohort does not allow us to follow individuals who have out-migrated from the site. Some of these individuals may have developed epilepsy and would not have been diagnosed in the study, though those lost to follow-up were generally younger and female.

In the Agincourt site, both males and females had similar remission rates, which were lower than those reported elsewhere[36]. It is likely that these lower remission rates are a result of the higher mortality rates seen in this cohort, especially amongst males. Remission rates for other seizure types may vary from our findings as we only explored remission in those people with ACE. The remission rate is based on the epidemiological modeling of the DISMOD II software package, which can be verified by long-term follow-up of the epilepsy cohort in Agincourt.

### Mortality

Convulsive epilepsies are known to be associated with higher mortality than non-convulsive epilepsies[17]. We found that people with ACE have a three times greater chance of dying than the general population. Mortality was especially pronounced amongst males 50+ years old with ACE. A large, proportion of these deaths were due to infectious causes rather than epilepsy, suggesting that ACE does not contribute directly to the higher mortality rates seen in older men. Yet the overall mortality rates found in this study are similar to other studies from Africa[19], including studies from areas endemic for parasitic risk factors[18], suggesting that ACE is associated with substantial mortality in rural South Africa.

Our findings suggest roughly 40% of all deaths in people with ACE are either directly or indirectly related to epilepsy. However, unlike findings from rural China where the majority of deaths were caused by injury (indirectly related to epilepsy)[37], we found that the majority of deaths related to epilepsy were associated with prolonged seizures. This suggests probable status epilepticus, which could likely be prevented by reducing the treatment gap and improving access to primary, and immediate, care. These findings are consistent with a recent study from rural Kenya[7] and a review of epilepsy mortality in Africa[19].

### Risk Factors for Mortality

Males with epilepsy were more likely to die than females. This might reflect the fact that women in this area tend to seek medical care earlier and more often[38].

While having HIV has been linked with poorer outcomes and increased mortality, we found that being HIV+ was not a risk factor for death in people with ACE. The majority of epilepsy-related (direct and indirect) deaths occurred in individuals who did not have HIV and who were older, while HIV+ individuals with epilepsy were found to die younger from causes not related to epilepsy.

Mortality due to ACE may have been underestimated in this study as a prevalence survey was used to identify cases of ACE, which includes people who have previously been diagnosed or experienced epilepsy. An incident cohort would provide a better mortality estimate as significant mortality is observed in people with epilepsy within the first 1–2 years after onset[17,35].

## Conclusion

We found the crude incidence of convulsive epilepsy in rural South Africa to be lower than other studies from Africa. While we found similar mortality rates in people with ACE to other African studies, we found higher mortality rates amongst older men. In general, we found that men develop epilepsy earlier and have a much higher risk of dying from it than females. Efforts aimed at further understanding what causes epilepsy in older people and developing interventions to reduce prolonged seizures are likely to reduce the overall burden of ACE in rural South Africa.

### Contributors

Members of The SEEDS writing group are: Agincourt HDSS, South Africa: Ryan Wagner, Rhian Twine, Myles Connor, F Xavier Gómez-Olivé, Mark Collinson, Kathleen Kahn, Stephen Tollman; Ifakara HDSS, Tanzania: Honratio Masanja, Alexander Mathew (deceased); Iganga-Mayuge HDSS, Uganda: Angelina Kakooza, George Pariyo, Stefan Peterson, Donald Ndyomughenyi; Kilifi HDSS, Kenya: Anthony K Ngugi, Rachael Odhiambo, Eddie Chengo, Martin Chabi, Evasius Bauni, Gathoni Kamuyu, Victor Mung’ala Odera (deceased), James O Mageto, Charles R Newton; Kintampo HDSS, Ghana: Ken Ae-Ngibise, Bright Akpalu, Albert Akpalu, Francis Agbokey, Patrick Adjei, Seth Owusu-Agyei; London School of Hygiene and Tropical Medicine, UK: Christian Bottomley, Immo Kleinschmidt; King’s College London, UK: Victor C K Doku; Swiss Tropical Institute, Switzerland: Peter Odermatt; University College London, UK: Brian Neville, Josemir W Sander, Steve White; National Institutes of Health, USA: Thomas Nutman; Centers for Disease Control and Prevention, USA: Patricia Wilkins, John Noh

## Supporting Information

S1 Table Age and sex of individuals lost to follow-up compared with those remaining in cohort, Agincourt 2008–12. (DOCX)

S2 Table Univariate analysis of factors associated with mortality in people with ACE, Agincourt 2012. (DOCX)

S3 Table Crude incidence of convulsive epilepsy by sex, Agincourt 2012. (DOCX)

S4 Table Incidence Rates of epilepsy expressed in 5-year/10-year age bands, Agincourt 2012. (DOCX)
