# Supplementary material for: Incidence, Remission and Mortality of Convulsive Epilepsy in Rural Northeast South Africa
Source: PLoS One. 2015 Jun 8;10(6):e0129097. doi: 10.1371/journal.pone.0129097 (PMC4459982; doi:10.1371/journal.pone.0129097)
Supplement: S1 Table — (DOCX) [file pone.0129097.s001.docx]

**Supplementary table 1:** Age and sex of individuals lost to follow-up compared with those remaining in cohort, Agincourt 2008-12

|  | **Lost to follow-up** | | **Present in 2012** | | **p-value** |
| --- | --- | --- | --- | --- | --- |
|  |  |  |  |  |  |
|  | *n=9534* | | n=72222 | |  |
| **Sex,** Female, %* | 5775 | 61% | 35554 | 49% | <0.0001 |
| **Age**, Mean, SD** | 19.5 | (15) | 25.5 | (19) | <0.0001 |
| **Age band*** | **n** | **%** | **n** | **%** |  |
| 0-5 | 1781 | 19% | 9588 | 13% |  |
| 6-12 | 1556 | 16% | 11352 | 16% |  |
| 13-17 | 1211 | 13% | 8817 | 12% | <0.0001 |
| 18-28 | 2938 | 31% | 16691 | 23% |  |
| 29-49 | 1680 | 18% | 16562 | 23% |  |
| 50+ | 368 | 4% | 9212 | 13% |  |
|  |  |  |  |  |  |
| *chi-squared test used to determine p-value | | | | | |
| ** Student t-test used to determine p-value | | | | | |
