# Supplementary material for: Incidence, Remission and Mortality of Convulsive Epilepsy in Rural Northeast South Africa
Source: PLoS One. 2015 Jun 8;10(6):e0129097. doi: 10.1371/journal.pone.0129097 (PMC4459982; doi:10.1371/journal.pone.0129097)
Supplement: S2 Table — (DOCX) [file pone.0129097.s002.docx]

| **Factor** | **Deaths** | **Person-years observed** | **Mortality Rate per 1000 (95%CI)** | | **Rate Ratios (95% CI)** | | **p-value** |
| --- | --- | --- | --- | --- | --- | --- | --- |
|  |  |  |  |  |  |  |  |
| **Current Age** |  |  |  |  |  |  |  |
| 0-5 | 1 | 56 | 17.8 | (2.5-125.9) | 1 | (n/a) | **0.002** |
| 6-12 | 3 | 142 | 21.1 | (6.8-65.6) | 1.2 | (0.1-11.5) |  |
| 13-17 | 1 | 123 | 8.1 | (1.1-57.6) | 0.5 | (0.0-7.3) |  |
| 18-28 | 4 | 240 | 16.6 | (6.2-44.4) | 0.9 | (0.1-8.4) |  |
| 29-49 | 9 | 384 | 23.4 | (12.2-45.0) | 1.3 | (0.2-10.4) |  |
| 50+ | 15 | 176 | 85.2 | (51.4-141.5) | 4.8 | (0.6-36.4) |  |
| **Sex** |  |  |  |  |  |  |  |
| Female | 10 | 548 | 18.3 | (9.8-33.9) | 1 | (n/a) | **0.038** |
| Male | 23 | 574 | 40.1 | (26.6-60.3) | 2.2 | (1.0-4.6) |  |
| **Education** |  |  |  |  |  |  |  |
| Yes | 16 | 606 | 26.4 | (16.2-43.1) | 1 | (n/a) | **0.020** |
| No | 12 | 188 | 63.9 | (36.3-112.6) | 2.4 | (1.1-5.1) |  |
| **Receiving income*** |  |  |  |  |  |  |  |
| Yes | 3 | 80 | 37.4 | (12.1-115.9) | 1 | (n/a) | 0.982 |
| No | 25 | 678 | 36.9 | (24.9-54.6) | 1.0 | (0.3-3.3) |  |
| **HIV status** |  |  |  |  |  |  |  |
| Negative | 16 | 615 | 26 | (15.9-42.5) | 1 | (n/a) | 0.371 |
| Positive | 5 | 122 | 41.2 | (17.1-98.9) | 1.6 | (0.6-4.3) |  |
| **Learning Difficulties** |  |  |  |  |  |  |  |
| Yes | 11 | 292 | 37.7 | (20.9-68.1) | 1 | (n/a) | 0.402 |
| No | 22 | 794 | 27.7 | (18.2-42.1) | 1.4 | (0.6-2.8) |  |
| **Age at Onset** |  |  |  |  |  |  |  |
| 0-5 | 6 | 351 | 17.1 | (7.7-38.1) | 1 | (n/a) | **0.009** |
| 6-12 | 4 | 201 | 19.9 | (7.5-52.9) | 1.2 | (0.3-4.1) |  |
| 13-17 | 2 | 111 | 18.1 | (4.5-72.2) | 1.1 | (0.2-5.2) |  |
| 18-28 | 3 | 167 | 17.9 | (5.8-55.6) | 1.0 | (0.3-4.2) |  |
| 29-49 | 10 | 188 | 53.1 | (28.6-98.7) | 3.1 | (1.1-8.5) |  |
| 50+ | 6 | 58 | 103.3 | (46.4-229.8) | 6.0 | (1.9-18.7) |  |
| **Visits Health Facility**** |  |  |  |  |  |  |  |
| Yes | 23 | 776 | 29.6 | (19.7-44.6) | 1 | (n/a) | 0.913 |
| No | 7 | 248 | 28.3 | (13.5-59.3) | 0.9 | (0.4-2.2) |  |
| **Seizure Frequency**** |  |  |  |  |  |  |  |
| Yearly | 11 | 410 | 26.9 | (14.9-48.5) | 1 | (n/a) | 0.770 |
| Daily | 0 | 21 | . | . | . | . |  |
| Weekly or Monthly | 21 | 598 | 35.1 | (22.9-53.9) | 1.3 | (0.6-2.7) |  |
| **Receiving treatment**** |  |  |  |  |  |  |  |
| Yes | 24 | 672 | 35.7 | (23.9-53.3) | 1 | (n/a) | 0.255 |
| No | 8 | 357 | 22.4 | (11.2-44.9) | 1.59 | (0.71-3.54) |  |

**Supplementary table 2:** Univariate analysis of factors associated with mortality in people with ACE, Agincourt 2012

*in individuals ≥18 years

**expressed as time-varying variables
