# Supplementary material for: Incidence, Remission and Mortality of Convulsive Epilepsy in Rural Northeast South Africa
Source: PLoS One. 2015 Jun 8;10(6):e0129097. doi: 10.1371/journal.pone.0129097 (PMC4459982; doi:10.1371/journal.pone.0129097)
Supplement: S3 Table — (DOCX) [file pone.0129097.s003.docx]

|  | **Person-Years Observed** | **Incident Cases of ACE** | **Crude Incidence (per 100,000)** | **95% CI** |
| --- | --- | --- | --- | --- |
|  |  |  |  |  |
| **Female** | 140652 | 24 | 17.1 | (11.4-25.5) |
| **Male** | 135748 | 24 | 17.7 | (11.9-26.4) |

**S3 Table.** Crude incidence of convulsive epilepsy by sex, Agincourt 2012
