# Supplementary material for: Incidence, Remission and Mortality of Convulsive Epilepsy in Rural Northeast South Africa
Source: PLoS One. 2015 Jun 8;10(6):e0129097. doi: 10.1371/journal.pone.0129097 (PMC4459982; doi:10.1371/journal.pone.0129097)
Supplement: S4 Table — (DOCX) [file pone.0129097.s004.docx]

**S4 Table.** Incidence Rates of epilepsy expressed in 5-year/10-year age bands, Agincourt 2012

| **Age band** | **Crude Incidence (per 100,000)*** | **95% CI** |
| --- | --- | --- |
|  |  |  |
| 0-4 | 29.9 | (7.5-119.7) |
| 5-14 | 17.5 | (9.7-31.6) |
| 15-24 | 17.3 | (9.8-30.5) |
| 25-34 | 13.3 | (6.4-28.0) |
| 35-44 | 15.1 | (6.3-36.3) |
| 45-54 | 27.3 | (12.2-60.7) |
| 55-64 | 21.0 | (6.8-65.1) |
| 65-74 | 12.1 | (1.7-85.9) |
| 75-84 | 18.6 | (2.6-131.9) |
